# Movement behaviours in paediatric cancer survivors during recovery and school weeks

**DOI:** 10.3389/fonc.2022.971805

**Published:** 2022-09-12

**Authors:** Tomáš Vyhlídal, Jan Dygrýn, Jana Pelclová, František Chmelík

**Affiliations:** Faculty of Physical Culture, Palacký University Olomouc, Olomouc, Czechia

**Keywords:** sedentary behaviour, accelerometery, physical activity, paediatric cancer survivors, recovery week, school week, summer camp

## Abstract

**Purpose:**

Paediatric cancer survivors (PCS) are a high-risk population, who can suffer from late effects of their treatment, such as metabolic syndrome, cardiovascular conditions, secondary tumours. Optimal movement behaviours (e.g., limited sedentary behaviour [SB] and sufficient physical activity [PA]) can reduce the side effects or avoid late effects of their treatment. The aims of this study were to analyse movement behaviours and meeting the recommendation of 60 minutes of moderate-to-vigorous physical activity (MVPA) a day in Czech PCS, and to compare their movement behaviours during recovery and school weeks in relation to gender, age, and cancer type.

**Methods:**

Twenty-six PCS aged 7-15 years in remission stage took part in the cross-sectional study. Movement behaviours were measured with Actigraph wGT3X+ accelerometers worn 24 hour/day for 20 consecutive days covering recovery week (13 days at recovery camp) and school week (7 days). Based on cancer types, the PCS were categorized into haematological malignancy or solid tumours group.

**Results:**

In the PCS, movement behaviours differed between recovery and school weeks. During recovery week, the PCS showed less SB (451.8 vs. 552.3 min/day, p < 0.001) and spent more time on light PA (350.3 vs. 255.1 min/day, p < 0.001), moderate PA (73.2 vs. 37.4 min/day, p < 0.001), and vigorous PA (10.3 vs. 4.0 min/day p < 0.001) than during school week. The PA recommendation was met by 77% (n = 20) PCS during recovery week, but only by 15% (n = 4) individuals during school week.

**Conclusions:**

The PCS recorded higher levels of PA and lower levels of SB during recovery week than during school week. If provided with appropriate conditions, PCS in the remission stage are able to reach the PA level recommended for the healthy population. Recovery week can be a suitable platform for gaining experience that PCS are able to meet the recommended PA level and could be an integral part of reconditioning and resocialization programmes for PCS after the completion of their treatment.

## Introduction

About four hundred new cases of oncologic diseases in children are diagnosed in the Czech Republic every year. According to the reports of the epidemiology of cancer diseases in childhood (1), almost 9500 cancer diseases were diagnosed in childhood between 1994 and 2016. Groups of leukaemic diseases and other haemato-oncological diagnoses were the most prevalent. Malignant diseases were more often diagnosed in boys. In the 1996-2016 period, this concerned 224 cases in boys and 186 cases in girls per year.

Paediatric cancer survivors (PCS) are a high-risk group who are very likely to experience consequences of their treatment at an older age, which can affect their condition adversely (2–5). For instance, nephrotoxicity, ototoxicity, and osteonecrosis belong among the effects of the treatment that can manifest themselves within five years after the treatment and prevail at a later age. Metabolic syndrome, cardiovascular conditions, secondary tumours/malignancies, conditions affecting the kidneys, diabetes mellitus, post-traumatic stress disorder, depression, and many others belong among the long-lasting late effects (6).

Bauman et al. (7) who focused on physical activities (PA) in paediatric oncology, state that regular PA can help mitigate or avoid unwanted effects of the treatment later. Thanks to regular PA, patients feel reduced tiredness, enhanced immunity, and better sleep and overall quality of life. Positive effects of PA in the field of paediatric oncology have been supported by other studies too (8–11). The importance of physical activity in this target group is also emphasised by the international expert group that has developed the International Paediatric Oncology Exercise Guidelines (iPOEG). These recommendations aim to promote individual behavioural and practice change (12).

Despite the benefits of regular PA, PCS are less physically active during the remission period than their peers (13, 14). Along with insufficient PA, elevated levels of sedentary behaviour (SB) represent another risk, which furthermore introduces another threat of chronic diseases (15). Existing literature suggests that movement behaviours may vary in different groups of PCS regarding their gender (16) or age (17) as opposed to their cancer type, which was not associated with different amounts of physical activity (18).

One of the means that can help improve the health of PCS are various forms of recovery camps. Positive effects on PCS related to their participation in these stays have been demonstrated, both in the psychological (19) and social fields (20, 21). Less attention was paid to the influence of recovery camps on movement behaviours. Self-reported data suggest that the camps may temporarily contribute to an increase in PA levels (22, 23). However, there are no known studies which have examined movement behaviours of PCS objectively during recovery camps and the following habitual school weeks.

Therefore, the aims of this study were [1] to analyse movement behaviours and compliance with the recommendation of 60 minutes of moderate-to-vigorous physical activity (MVPA) a day in Czech PCS, and [2] to compare their movement behaviours during recovery (RW) and school weeks (SW) in relation to gender, age, and cancer type.

## Methods

### Design and participants

PCS aged 7-15 years during the remission took part in the research study. The PCS underwent active treatment at the Department of Paediatric Oncology at the University Hospital Brno, which is one of the two chief centres for paediatric oncology in the Czech Republic. The participants in the study were Czech citizens and were diagnosed with an oncologic disease in accordance with the International Classification of Childhood Cancer (ICCC). The remission period of the participants is defined as a period of at most five years from the end of the maintenance therapy for cancer. Because of the large number of distinct types of oncologic diseases, we decided to split them into: a) haematological malignancy and b) solid tumours. This division is supported e.g. by authors Rehorst-Kleinlugtenbelt et al. (18) and Koçak et al. (24). Furthermore, the participants were stratified by gender and age. Two age categories were 7–11 years (middle childhood) and 12–15 years (early adolescence). The inclusion criteria for participation in the study were age between 6 and 15 years (target age group for participation in the RW), < 5 years remission period, and completion of active treatment at the Department of Paediatric Oncology at the University Hospital Brno. Exclusion criteria included disability or health condition unrelated to the treatment.

### Recruitment of participants

**Figure 1:**
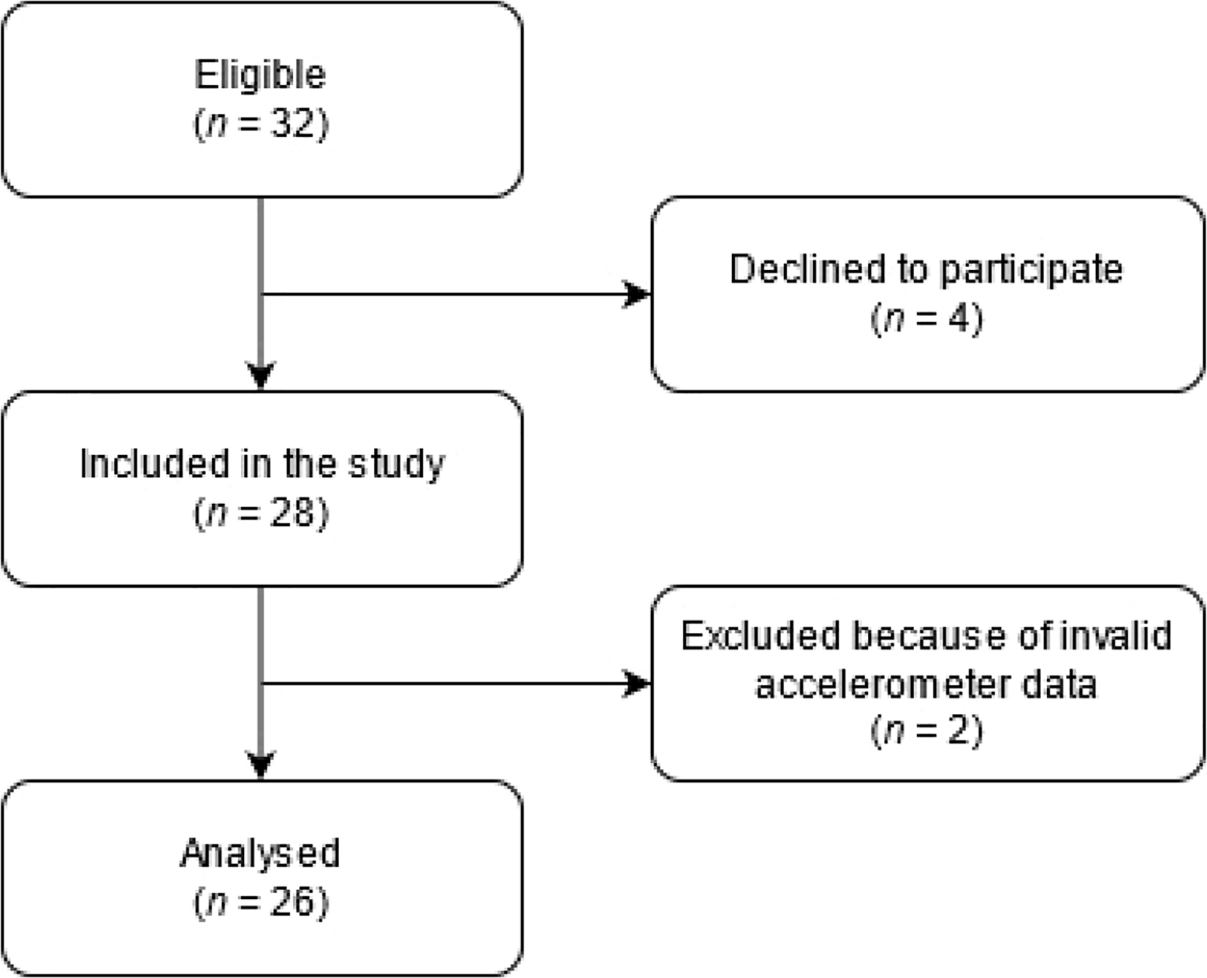
Flowchart of the study.

Based on the inclusion criteria, legal guardians of the research participants were approached, which was carried out by the main organizer of the RW – the KRTEK Foundation for paediatric oncology. The total number of participants eligible for the study who were addressed to take part was 32 (**Figure 1**). Four children expressed no interest in getting involved in the pilot research. The data of two participants could not be assessed because of insufficient time spent wearing the device. Overall, the PA and SB of 26 PCS during remission were assessed (the dataset is available in the **Supplementary Material**).

### Data collection

The data collection took place in the period between 19th August and 9th September 2018 and lasted for 20 days. The data was collected during the RW which were organized in the form of recovery summer camp by the KRTEK Foundation for paediatric oncology, and carried out for PCS treated at the Department of Paediatric Oncology at the University Hospital Brno between 18th and 31st August 2018. Because both PCS and their non-diagnosed siblings or peers take part, these RW have an integrative nature. Awareness of the significance of PA and the promotion of an active lifestyle of all the participants are the key pillars of the programme of these RW, as they include various types of PA (team games, hiking, water activities, dance activities, etc.) and do not differ from regular camps for healthy children. If necessary (secondary effects of treatment, acute health condition), all the PAs are modified in such a way that all the participants can engage in them. These RW have a defined daily schedule and programme regardless of the diagnosis, gender, or age of the participants. The programme starts at 7:30 a.m. by waking up and ends at 9:00 p.m. when the participants go to bed. As there is no systematic support for this target group at the national level in the Czech Republic, these RW are primarily organized by foundations or endowment funds.

After RW, the data collection took place until 9th September 2018 and focused on SW. The participants received the monitoring devices on the first day of RW. Data was collected from the second day of RW. The study participants and their parents or guardians were notified of the study two weeks before the research began. Parents’ or guardians’ consent was required for participation in the study. The study was approved by the Ethics Committee of the Faculty of Physical Culture, Palacký University Olomouc under reg. no. 48/2018.

### Measures

#### Movement behaviours

The SB and PA were measured using the Actigraph wGT3X+ accelerometer (ActiGraph LLC, Pensacola, FL, USA). The participants were asked to wear the device for 20 consecutive days. The devices were worn on the wrist of the non-dominant arm for the entire monitoring period, except while having a bath or shower. The accelerometers were set up using the Actilife software (ActiGraph LLC, Pensacola, FL, USA) to record the raw data on three axes. The default sampling frequency 30 Hz was chosen as a compromise to achieve data to be sufficient for body movements classification (25) and extend battery life for the entire measurement period (https://actigraphcorp.com/actigraph-wgt3x-bt/). To estimate the volume and intensity of movement behaviours, we used the R package GGIR (v. 1.10-4, https://cran.r-project.org/web/packages/GGIR/). A detailed description of the methods is provided elsewhere (26). Time in SB and PA intensities was assigned using previously published thresholds for 5-sec epochs: <36 miligravitational units (mg) for SB; ≥ 36 mg and <201 mg for light PA (LPA); ≥ 201 mg and <707 mg for moderate PA (MPA); ≥707 mg for vigorous PA (VPA) (27, 28). The participants were required to wear the accelerometers for at least 16 hours a day. If the device wear time was shorter, the record of such a day was excluded from the analyses. Participants with at least 4 valid days (including at least 1 weekend day) in both RW and SW were included in the analysis (26).

To enable a comparison with the new guidelines of the World Health Organization for PA and SB for children and adolescents (5-17 years), including children and adolescents with health impairments, a cut-off point of 60 minutes of MVPA per day was set. MVPA is characterized as a health-enhancing PA performed at the intensity of >3.0 metabolic equivalent (29). In contrast, SB is any behaviour defined as any waking behavior in a sitting, reclining or lying posture with an energy expenditure of ≤1.5 metabolic equivalent (30).

#### Body Mass Index

The Body Mass Index (BMI) was calculated by dividing the child’s weight (kg) by height (m) squared. The participants’ body weight and height were measured first day of the RW using a Tanita™ calibrated digital scale (UM-075 type; Tanita Corporation, Tokyo, Japan) and the Leicester height measure.

#### Information on treatment

Clinical data, including the type of oncologic disease and length of treatment, was retrieved by a questionnaire survey from the parents or guardians of the study participants.

### Statistical analysis

The IBM SPSS Statistics 25 (IBM SPSS, Inc. Chicago, IL, USA) software was used to process the data from the monitoring. The characteristics of the study sample are presented using descriptive statistics (median and interquartile range). To compare the study groups (age, gender, type of oncologic disease), the Mann-Whitney U Test was used. To assess the differences between the RW stay and SW, a non-parametric Wilcoxon signed-rank test for paired data was used. A chi-squared test was used for the comparison of proportions. The level of statistical significance was set at α=0.05. The effect size coefficients were interpreted as follows: 0.2 ≤ d < 0.5 – small effect size, 0.5 ≤ d < 0.8 – medium effect size, and d ≥ 0.8 – large effect size.

## Results

**Table 1** displays the basic descriptive characteristics of the study sample.

**Table 1:** Descriptive characteristics of the study sample (n = 26).

	Age (years)			Body height (cm)		Body weight (kg)		BMI (kg/m2)	
	N	Mdn	IQR	Mdn	IQR	Mdn	IQR	Mdn	IQR
**Gender**									
Boys	12	12.4	3.6	156.4	30.1	39.6	16.5	17.4	3.4
Girls	14	12.1	3.7	150.2	14.7	47.7	21.3	20.3	6.6
**Age**									
7-11 years	12	10.1	2.1	140.6	12.1	35.3	10.3	18.1	3.7
12-15 years	14	13.2	2.3	160.7	13.1	53.0	19.7	20.0	5.7
**Type of disease**									
Haematological malignancy	14	12.0	3.5	154.1	19.9	49.2	23.9	20.3	5.0
Solid tumours	12	12.5	4.2	146.0	26.0	35.5	16.4	17.2	4.0
**TOTAL**	26	12.1	3.5	151.9	20.5	40.3	18.9	18.8	4.7

N, number of participants; Mdn, Median; IQR, Interquartile range.

Descriptive statistics of movement behaviours and rates of meeting the recommendation of 60 minutes of MVPA daily during RW and SW are presented in **Table 2**. We observed significant differences in PCS between RW and SW. The PCS accumulated more minutes of LPA, MPA, and VPA and fewer minutes of SB during RW. In addition, more of them met the recommended PA level.

**Table 2:** Movement behaviours and meeting of recommended PA level in paediatric cancer survivors during recovery and school weeks.

	Recovery weeks		School weeks		Difference	
	Mdn	IQR	Mdn	IQR	p-value	d
**Movement behaviours**						
SB (min/day)	451.8	105.9	552.3	112.4	<0.001	1.64
LPA (min/day)	350.3	62.0	255.1	97.3	<0.001	1.68
MPA (min/day)	73.2	50.6	37.4	21.8	<0.001	1.74
VPA (min/day)	10.3	13.1	4.0	6.6	<0.001	1.75
**Meeting recommendation**						
60 min of MVPA/day (N, %)	20	77	4	15	<0.001	

Mdn, Median; IQR, Interquartile range; d, effect size coefficient; SB, sedentary behaviour; LPA, light physical activity; MPA, moderate physical activity; VPA, vigorous physical activity; MVPA, moderate-to-vigorous physical activity.

More detailed analysis accounting for gender, age, and cancer type is shown in **Table 3**. All the study subgroups were more physically active and less sedentary during RW than during SW. Compared to SW, a higher number of the participants also achieved the PA recommendation during RW.

**Table 3:** Movement behaviours and meeting recommended PA level in paediatric cancer survivors during recovery and school weeks in different gender, age, and cancer type groups.

	Recovery weeks		School weeks		Difference	
	Mdn	IQR	Mdn	IQR	p-value	d
**Gender**						
**Movement behaviours boys (n=12)**						
SB (min/day)	493.9	98.2	577.9	127.4	0.006	1.59
LPA (min/day)	336	62.3	219.6^b^	41.8	0.002	1.77
MPA (min/day)	62.5	55.5	34.2	18.1	0.003	1.72
VPA (min/day)	11.3	15.2	43.8	6.7	0.002	1.77
**Meeting recommendation**						
60 min of MVPA/day (N, %)	9	75	1	8	0.001	
**Movement behaviours girls (n=14)**						
SB (min/day)	419.9	110.3	534.8	107.5	0.001	1.73
LPA (min/day)	362.8	58.9	293.0^b^	82.6	0.004	1.56
MPA (min/day)	85.2	51.1	38.4	27.4	0.001	1.76
VPA (min/day)	9.8	13.1	4	5.9	0.001	1.76
**Meeting recommendation**						
60 min of MVPA/day (N, %)	11	79	3	21	0.002	
**Age**						
**Movement behaviours Age 7-11 (n=12)**						
SB (min/day)	413.3	121.7	497.8^b^	74.3	0.015	1.41
LPA (min/day)	357.7	56.4	299.1^b^	87.7	0.003	1.72
MPA (min/day)	95.2	61.2	43.9^b^	29.6	0.003	1.72
VPA (min/day)	17.4^a^	17	5.3^b^	8.9	0.002	1.77
**Meeting recommendation**						
60 min of MVPA/day (N, %)	9	75	4	33^b^	0.043	
**Movement behaviours Age 12-15 (n=14)**						
SB (min/day)	488.4	74.3	609.9^b^	110.5	0.001	1.76
LPA (min/day)	338.5	64.2	217.3^b^	67.4	0.002	1.7
MPA (min/day)	66.2	28.7	30.5^b^	18.5	0.001	1.76
VPA (min/day)	9.8^a^	11.4	2.2^b^	3.5	0.001	1.76
**Meeting recommendation**						
60 min of MVPA/day (N, %)	11	79	0	0^b^	<0.001	
**Cancer type**						
**Movement behaviours Haematological malignancy (n=14)**						
SB (min/day)	476.6	99.6	534.8	92	0.005	1.49
LPA (min/day)	347.6	61	274.7	114.1	0.003	1.59
MPA (min/day)	74.1	38.8	38.4	21.6	0.001	1.73
VPA (min/day)	8.7	8.7	4	7.1	0.001	1.76
**Meeting recommendation**						
60 min of MVPA/day (N, %)	10	79	2	14	0.001	
**Movement behaviours Solid tumours (n=12)**						
SB (min/day)	428.3	131.7	592.2	140.7	0.002	1.77
LPA (min/day)	353.1	67	226.5	82	0.002	1.77
MPA (min/day)	73.2	56.8	36.2	22	0.002	1.77
VPA (min/day)	17.6	12.9	3.6	6.3	0.002	1.77
**Meeting recommendation**						
60 min of MVPA/day (N, %)	9	75	2	17	0.005	

Mdn, Median; IQR, Interquartile range; d, effect size coefficient; SB, sedentary behaviour; LPA, light physical activity; MPA, moderate physical activity; VPA, vigorous physical activity; MVPA, moderate-to-vigorous physical activity. ^a^significant difference between paediatric cancer survivor groups in recovery weeks.

^b^significant difference between paediatric cancer survivor groups in school weeks.

No significant differences in PA, SB, and the rate of meeting the PA recommendation were found between the boys and girls during RW. This was similar for SW with one exception, as the girls recorded significantly more LPA (p = 0.013; d = 0.96) than the boys.

A comparison of the age groups showed that the younger PCS accumulated more VPA during RW (p = 0.041; d = 0.81), compared with the older participants. There were significant differences between the age groups in all the parameters that were monitored during SW, though. The younger PCS recorded more minutes of LPA (p = 0.017; d = 0.93), MPA (p = 0.036; d = 0.83), as well as VPA (p = 0.013; d = 0.97) and fewer minutes of SB (p < 0.001; d = 1.31), and more of them (p = 0.022) met the recommended PA level than was the case for their older peers. None of the older PCS participants met the PA recommendation in SW.

We observed no significant differences in PA, PA recommendation, and SB between groups of PCS with distinct types of cancer diagnosis during RW. A similar result also held true for SW.

## Discussion

The main findings of the present study showed that the movement behaviours of the PCS differed between RW and SW. The PCS had more minutes of LPA, MPA, and VPA and a shorter time of SB during RW, compared with SW. In addition, a lower proportion of the PCS reached the recommended level of daily MVPA during SW compared to RW.

We were unsuccessful in our attempts to find a similar study that compares the movement behaviours of PCS in two distinct environments in the available literature. A comparison with the findings of Braam et al. (31) who monitored SB and PA during treatment or one year after its end at most, implies that the PCS in our study had higher levels of PA and lower levels of SB. The results of our study could indicate that one could expect a return to a higher level of PA and a lower level of SB after a longer time from the end of treatment.

Our findings of higher PA and lower SB in RW were confirmed regardless of the age, gender, and cancer type of the PCS. Our comparison of movement behaviours indicators revealed a significant difference in LPA during SW, with the girls accumulating more minutes of PA of light intensity than the boys. This finding is in contrast with the results published previously (16, 22), which showed higher levels of PA rather in boys.

With regard to age, the differences were particularly apparent in SW. The younger age category (7-11 years) recorded significantly higher levels of PA. A decrease in PA with age is supported by a study by Farooq et al. (17), who reported that the level of PA in the population declines, regardless of gender, starting from 6-7 years of age. A similar decrease in PA among PCS is also apparent in our study.

We found no differences in PA according to the type of cancer disease – haematological malignancy and solid tumours. We can assume that the type of oncologic diagnosis does not affect PA in PCS in the remission period, unless secondary or late effects of treatment occur. This finding is confirmed by Rehorst-Kleinlugtenbelt et al. (18).

Significant differences between RW and SW are further supported by results that compare the rates of meeting the PA recommendation in the two periods of monitoring. The proportion of those who met the recommended PA level during RW suggests that PCS in the remission stage are able to comply with the PA recommendations for the healthy population. The results of other studies from Switzerland (32) and Australia (33) support this finding. On the basis of self-reported data (questionnaire survey, PA diary), their authors concluded that PCS who finished their treatment can meet the PA recommendation (74% in Switzerland and 55% in Australia). The decline in PA during SW, which represents a common habitual movement routine, can be considered a ‘red flag’. Thus, it is necessary to focus on SW systematically, and target eventual interventions for the promotion of an active lifestyle specifically to SW.

Our study suggests that, if appropriate conditions are in place, PCS in the remission stage are able to reach the level of PA recommended for the healthy population. The strengths of this study include the objectively measured PA and SB data and, in particular, the long period of monitoring. The quantification of PA and SB in two completely different environments offers a new insight into movement behaviours in PCS. It is also the first study from the Czech Republic that takes gender, age, and cancer type into account.

The small study sample and the specific conditions of one RW may pose a limit to the generalizability of the study findings. It is uncertain to what extent our results related to the RW can be transferred to other PCS settings and services. Therefore, further research is required to help better understand how recovery should be designed in routine aftercare to become an acceptable, feasible, effective, and sustainable way to improve patient outcomes.

## Conclusions

Our study suggests that PCS during remission can achieve the level of PA recommended for the healthy population. Future studies should focus on the elimination of barriers that lead to insufficient PA and excessive sitting of PCS especially during SW. Concurrently, the present study showed that RW can be a suitable platform for gaining experience that PCS are able to meet the recommended PA level. RW could be an integral part of reconditioning and resocialization programmes for PCS after they complete the treatment.

## Data availability statement

The dataset is included in the **Supplementary Material**. Further inquiries can be directed to the corresponding author.

## Ethics statement

The study was reviewed and approved by Ethics Committee of Palacký University Olomouc (No. 48/2018). Written informed consent to participate in this study was provided by the participants’ legal guardian/next of kin.

## Author contributions

All authors contributed to the study conception and design. Material preparation, data collection, and initial analysis were performed by TV. The first draft of the manuscript was written by TV and all authors commented on and critically revised all versions of the manuscript. All authors read and approved the final manuscript.

## Funding

This study was funded by Internal grant of Palacký University Olomouc (IGA_FTK_2019_011).

## Conflict of interest

The authors declare that the research was conducted in the absence of any commercial or financial relationships that could be construed as a potential conflict of interest.

## Publisher’s note

All claims expressed in this article are solely those of the authors and do not necessarily represent those of their affiliated organizations, or those of the publisher, the editors and the reviewers. Any product that may be evaluated in this article, or claim that may be made by its manufacturer, is not guaranteed or endorsed by the publisher.
